# The Metaverse as a virtual form of data-driven smart urbanism: platformization and its underlying processes, institutional dimensions, and disruptive impacts

**DOI:** 10.1007/s43762-022-00051-0

**Published:** 2022-08-12

**Authors:** Simon Elias Bibri, Zaheer Allam, John Krogstie

**Affiliations:** 1 Department of Computer Science, Norwegian University of Science and Technology, Sem Saelands veie 9, NO–7491 Trondheim, Norway; 2 Department of Architecture and Planning, Norwegian University of Science and Technology, Alfred Getz vei 3, Sentralbygg 1, 5th floor, NO–7491 Trondheim, Norway; 3 Chaire Entrepreneuriat Territoire Innovation (ETI), IAE Paris—Sorbonne Business School, Université Paris Panthéon-Sorbonne, 75013 Paris, France; 4 Live+Smart Research Lab, School of Architecture and Built Environment, Deakin University, Geelong, VIC 3220 Australia

**Keywords:** Metaverse, Data-driven smart urbanism, Hyper-connectivity, Datafication, Algorithmizaton, Platformization, COVID-19 pandemic, Surveillance, Governance, Privacy, Ethics

## Abstract

The emerging phenomenon of platformization has given rise to what has been termed "platform society,“ a digitally connected world where platforms have penetrated the heart of urban societies—transforming social practices, disrupting social interactions and market relations, and affecting democratic processes. One of the recent manifestations of platformization is the Metaverse, a global platform whose data infrastructures, governance models, and economic processes are predicted to penetrate different urban sectors and spheres of urban life. The Metaverse is an idea of a hypothetical set of “parallel virtual worlds” that incarnate ways of living in believably virtual cities as an alternative to future data-driven smart cities. However, this idea has already raised concerns over what constitutes the global architecture of computer mediation underlying the Metaverse with regard to different forms of social life as well as social order. This study analyzes the core emerging trends enabling and driving data-driven smart cities and uses the outcome to devise a novel framework for the digital and computing processes underlying the Metaverse as a virtual form of data-driven smart cities. Further, it examines and discusses the risks and impacts of the Metaverse, paying particular attention to: platformization; the COVID-19 crisis and the ensuing non-spontaneous "normality" of social order; corporate-led technocratic governance; governmentality; privacy, security, and trust; and data governance. A thematic analysis approach is adopted to cope with the vast body of literature of various disciplinarities. The analysis identifies five digital and computing processes related to data-driven smart cities: digital instrumentation, digital hyper-connectivity, datafication, algorithmization, and platformization. The novelty of the framework derived based on thematic analysis lies in its essential processual digital and computing components and the way in which these are structured and integrated given their clear synergies as to enabling the functioning of the Metaverse towards potentially virtual cities. This study highlights how and why the identified digital and computing processes—as intricately interwoven with the entirety of urban ways of living—arouse contentions and controversies pertaining to society’ public values. As such, it provides new insights into understanding the complex interplay between the Metaverse as a form of science and technology and the other dimensions of society. Accordingly, it contributes to the scholarly debates in the field of Science, Technology, and Society (STS) by highlighting the societal and ethical implications of the platformization of urban societies through the Metaverse.

## Introduction

While the idea of the Metaverse has been around for three decades as a speculative fiction narrative where users are represented as avatars in unconnected virtual spaces, it is until recently that it came to the public fore with the rebranding of Facebook into “Meta” and other platform providers. The Metaverse has been made possible by the rapid pace of progress in the development of the core enabling technologies, notably Artificial Intelligence (AI), Big Data, the Internet Things (IoT), Edge Computing, Blockchain, Digital Twins (DT), Virtual Reality (VR), Augmented Reality (AR), Mixed Reality (MR), and high-speed 5G networks. While these technologies are not of equal importance in terms of enabling the Metaverse as a “sophisticated” computing platform, their convergence has expedited the integration of the independent virtual spaces owned by many different platform companies. Meta is one of the globally operating platform companies. Platforms have become crucial for understanding future-focused or envisioned urbanism in emerging data-driven smart cities (Allam et al. 2022a). Smart urbanism and platform urbanism are interrelated as approaches to urban development in that the latter originated in the multifaceted emergence and rapid development of the former over the past two decades. Generally, smart urbanism is understood as a model of urban development focused on the use of big data analytics, digital flows, and networked technologies (Bettencourt, 2014; Kitchin, 2014). These aspects of smart urbanism tend to capture the nature of platform urbanism as a manifestation of the process and practice of platformization. In short, platform urbanism is seen as an evolution of smart urbanism (Han and Hawken 2018). Caprotti et al. (2022) define platform urbanism as a “novel set of digitally enabled socio-technological assemblages rooted in the urban, which enables the emergence of new social and material relationships including intermediations and transactions.”

Moreover, research and development of the Metaverse has become a key trend in smart urbanism in terms of the design of believably virtual cities based on large-scale data-driven AI systems (Bibri, 2022). This relates to what has been termed “virtual urbanism” or “augmented urbanism” (Gordon and Manosevitch, 2011; Sirc, 2001; Wilkins and Stiff, 2019) with respect to the application of urban planning, urban design, and urban geography to the design of virtual and augmented urban spaces. In studying the effects of the emergence of virtual cities have on their perceptions compared to real-world cities, Hemmati (2022) found that the Metaverse can create more believable images than reality. As an envisioned form of virtual urbanism, the Metaverse denotes “a set of virtual spaces where you can create and explore with other people who are not in the same physical space as you. You will be able to hang out with friends, work, play, learn, shop, create, and more” (Bosworth and Nick, 2021). The whole idea of the Metaverse as a form of scientific and technological development relates to the long-established debate on the role of science and technology in social progress (see, e.g., Cutcliffe, 2000; Cutcliffe, 2001; Jasanoff and Kim, 2009; Volti, 2001). In the light of the negative impacts that the social media platforms owned by Meta have had on urban society, coupled with the plethora of thorny issues they have raised, over the last two decades, the Metaverse will likely fail to justify scientific and technological development and investment in the sense of equating science and technology with societal progress (Bibri, 2022). That is, with the advancement of the conditions of urban society and how people live in it based on prevailing norms, values, beliefs, and goals. Societal progress entails that the current conditions of society are improved compared to the past, and that these conditions are envisaged to be better than those of the present (Noll, 2014).

The Metaverse depicts the peculiar characteristics of ways of living in data-driven smart cities of the future. Urbanism denotes “the distinctive features of the experience of everyday life in cities” (Bridge, 2009, p. 106), which are being highly responsive to a form of data-driven smart urbanism and platform urbanism based on AI and analytics systems with regard to urban services and urban governance. The radical expansion of the granularity, range, and magnitude of urban big data and data-intensive compute algorithms combined with the onset of AI techniques has become compounded by the COVID-19 pandemic. One implication of this is that this crisis has induced big tech companies to look for new ways to cater for the growing demand in speed, scale, and extension of AI-software systems towards large-scale data-driven AI systems given their potential for enabling “sophisticated” forms of governance. Smart governance has been criticized because it is strongly driven by the interests and agenda of high-tech companies and large corporations as well as the associated government policies (e.g., Grossi and Pianezzi, 2017; Hollands, 2015).

Moreover, the “new normal” established in the aftermath of the COVID-19 pandemic has resulted in an abrupt large-scale digital transformation of urban society, a process of digitization and digitalization that is in turn paving the way for merging virtual reality and physical reality in the context of data-driven smart cities. This merger requires the intensification of the datafication, algorithmization, and platformization of both socializing, working, learning, playing, travelling, shopping, and so on, as well as the social organization resulting from these interactions and activities (Bibri and Allam, 2022a). This epitomizes the core of the Metaverse vision in terms of its ultimate goal to virtualize ways of living and working in urban society. This concept refers to the social organization resulting from social interaction as an essential aspect of social life, the ways in which people act with other people and react to their ways of acting, as well as the interaction of people with the physical environment (Bibri, 2022). With reference to smart cities, however, Calvo (2020) argues that the escalating digital and computing trends are, either intentionally or unintentionally, associated with highly corrosive consequences for urban society. In addition, smart city systems “are often based on technological orthodoxies which are conceptually and empirically shallow” (Viitanen and Kingston, 2015). In other words, smart cities are cast as “bounded, knowable, and manageable…that can be steered and controlled in mechanical, linear ways” (Kitchin, 2016, p. 11). Consequently, numerous studies have addressed, from a variety of perspectives, the potential risks and other negative implications of smart cities (e.g., technocratic reductionism, technocentricity, governance corporatization, technological lock-ins, surveillance, privacy loss, mind control, democratic decay backsliding) and the ramifications of the infiltration of socially disruptive technologies into the fabric of urban life and urban environment (see Bibri, 2021a, 2021b for a detailed overview). In view of the above, the downsides of the Metaverse as a virtual form of data-driven smart urbanism or platform urbanism remain unavoidable. This inescapable situation, especially the potential issues and risks that are not immediately obvious but easily encountered, are most likely to affect the social life and social order of urban society in terms of social structures and institutions, social relations, social interaction and behavior, and cultural norms and values.

Against the preceding background, this study analyses the emerging trends enabling and driving data-driven smart cities and uses the outcome to devise a novel framework for the digital and computing processes underlying the Metaverse as a virtual form of data-driven smart cities. Further, it examines and discusses the risks and impacts of the Metaverse, paying particular attention to: platformization; the COVID-19 crisis and the ensuing non-spontaneous "normality" of social order; corporate-led technocratic governance; governmentality; privacy, security, and trust; and data governance.

This study is structured as follows: Section 2 presents a survey of related work in terms of the state–of–the–art research. Section 3 introduces, outlines, and justifies the research methodology adopted in this study, Section is 4 presents the results of the thematic analysis. This study ends, in Section 5, with discussion and conclusion.

## Related Work

The permeation of urban society by digital platforms in regard to data infrastructures, economic processes, and governance models has provided the globally operating platform companies with the opportunity to leapfrog their way of thinking and devising complex platforms by courtesy of data-driven smart urbanism and platform urbanism. One of such platforms is the Metaverse, a gigantic ecosystem application that is fuelled by the most innovative computing and immersive technologies. Given the current development stage of the Metaverse as a global platform being launched in 2021 by Meta, research in this area tends to focus mainly on two strands, which are typical to the advent of new socially disruptive technologies. The first strand is concerned with the state-of-the-art and technical aspects of the Metaverse in terms of computing technologies, immersive technologies, ecosystems, developments, trends, applications, opportunities, grand challenges, open issues, research agenda, roadmapping, and so on. One of the first studies that was conducted in this regard after the announcement of the Metaverse is the comprehensive state-of-the-art review by Lee et al. (2021). This focuses on the technologies that fuel the “Digital Big Bang” from the Internet and XR to the Metaverse, which support its ecosystem as a gigantic application. In addition to this detailed framework, the authors cover a plethora of other topics, as well as propose a research agenda and highlight the grand challenges associated with the development of the Metaverse. While the architecture proposed by Lee et al. (2021) consists of two key layers, Duan et al. (2021) propose a three-layer architecture for the Metaverse from a macro perspective: infrastructure, interaction, and ecosystem. Dhelim et al. (2022) address the state-of-the-art architecture for the Metaverse applications. The authors argue that it relies on a cloud-based approach for avatar physics emulation and graphics rendering computation, a centralized design that is unfavorable due to its several drawbacks caused by the long latency required for cloud access. To address this issue, they propose a Fog-Edge hybrid computing architecture that leverage an edge-enabled distributed computing paradigm. In such architecture, edge devices computing power is utilized to fulfil the required computational cost for heavy tasks, such as computation of 3D physics in virtual simulation and collision detection in virtual universe.

Similar to Lee et al. (2021), but different to their approach and with less detail, Mystakidis (2022) offers a comprehensive analysis of the extant literature, identifying current gaps or problems. The author discusses a number of topics of the Metaverse as a multiuser environment merging physical reality with digital virtuality, including XR and related concepts, multimodal Metaverse interactions, limitations of 2D learning environments, a brief history of virtual media and XR technologies, and Metaverse contemporary development. By way of conclusion, the author states that the Metaverse can enable world-wide participation on equal footing, unbound by geographical restrictions. Expanding on the history of virtual media, Duan et al. (2021) journey toward a historical and novel Metaverse. As regards the applications of the Metaverse, Taylor and Soneji (2022) examine how visualization can leverage the Metaverse in bioinformatics research and the advantages and disadvantages of this technology. Worth noting is that the applications of the Metaverse span a plethora of domains of urban society given its scope of use as a virtual form of data-driven smart urbanism and platform urbanism. Speaking of visualization, it is one of the several areas that is united by the rapidly evolving field of immersive analytics, in addition to immersive environments and human-computer interaction. This is expressed by Ens et al. (2021) who, in their study, present seventeen key research challenges aiming to coordinate future work by providing a systematic roadmap of current directions. The authors also provide impending hurdles in this area to facilitate productive and effective applications for immersive analytics. However, most of the aforementioned studies tend largely to focus on one or a few aspects of the technical strand of the Metaverse, lacking a more holistic perspective on the topic with respect to the historical embeddedness of science and technology, the socially constructed nature of science and technology, the social conditions and institutional structures shaping science and technology, and the societal and ethical implications of science and technology, and so on. In an attempt to address this gap in his recent study which is positioned within science of science, (Bibri, 2022) analyzes the complex interplay between the Metaverse as a form of science and technology and the wider social context, focusing on the intertwined factors underlying its materialization, expansion, success, and evolution, as well as the key contentions, bottlenecks, and uncertainties that have direct implications for its realization and acceptance. This study shows that the Metaverse raises serious issues and concerns related to social exclusion, marginalization, hive mentality, privacy erosion, surveillance, control, democratic backsliding, and dystopianism.

Given its focus, the above study relates to the second strand of research within the area of the Metaverse, which is concerned with its risks and other negative implications, engaging critically with the underlying core enabling technologies from a variety of perspectives. It is important to note that some of these implications have also been covered by some of the previous studies, but more or less from a computational perspective. For example, Lee et al. (2021) discuss a number of influencing design factors, including fairness, cyberbullying, device acceptability, avatar acceptability, and privacy threats, and argue that these will determine the sustainability of the Metaverse. Gurov and Konkova (2022) focus on the Metaverse for human or human for Metaverses by researching the grounds on which big tech companies seek to transform the mankind way of life and the nature of the “human,” based on the idea of the Metaverse. Using a comparative analysis, the authors highlight the opportunities and threats that the Metaverse pose for humanity in the conditions of the uncontrolled technological development. They conclude that forming and disseminating a new socio-humanitarian rationality is a necessary condition for the successful development of the Metaverse, predicated on the assumption that this approach will ensure control over the actions and activities of big tech companies. In this line of thinking, Rosenberg (2022) discusses the regulation of the Metaverse as a roadmap, outlining the dangers of the Metaverse along with proposals for regulation. Bibri and Allam (2022b) question and challenge the Metaverse through the prism of the logic of surveillance capitalism, focusing on how and why the practices of the governance of urban society are bound to be undemocratic and unethical. However, none of these studies has addressed the link between the Metaverse and data-driven smart urbanism from a conceptual perspective, nor the disruptive impacts of what underlie this relationship as an outcome of the process and practice of platformization and its institutional dimensions. The recent study conduced by Hemmati (2022) rather deals with the Metaverse as an urban revolution in regard to its effect on the perceptions of urban audience. The author found that the media seeks to create a purposeful image of reality in the minds of the audience, and the Metaverse can create more believable images than reality thanks to immersive technologies.

## Methodology: Thematic Analysis

This study assumes that there are trends and processes that repeat themselves and drive and underlie the digital architecture of computer mediation associated with the Metaverse as a virtual form of data-driven smart urbanism. Therefore, it uses a qualitative method to identify this architecture and its driving trends and underlying processes as concepts and, eventually, to identify the concepts behind them. In a broad sense, qualitative studies ultimately aim to describe and explain a pattern of relationships, a process that requires a set of conceptually specified categories (Mishler, 1990). The qualitative “tactics” to be used to generate meanings from a diverse empirical and theoretical material (Miles and Michael Huberman, 1994) relate to thematic analysis. Following these tactics, a thematic analysis has been designed for identifying the architecture and its digital and computing concepts and for conceptualizing the theoretical base behind these components. Thematic analysis is particularly, albeit not exclusively, associated with the analysis of textual material. In this respect, it emphasizes identifying, analyzing, interpreting, and reporting themes, i.e., important patterns of meaning within qualitative data. Worth pointing out is that, as suggested by Braun and Clarke (2006), thematic analysis is flexible in terms of theoretical and research design given that it is not dependent on any particular theory or epistemology: multiple theories can be applied to this process across a variety of epistemologies. However, this flexibility can lead to inconsistency when developing themes derived from the qualitative data (Holloway and Todres, 2003). Also, there is no one accurate interpretation of these data, interpretations reflect the positioning of the researcher.

As an inductive analytical technique, thematic analysis involves discovering patterns, themes, and concepts in the qualitative data that include interdisciplinary and transdisciplinary literature. As such, it allows these data to determine the set of themes to be identified, justified in this context by the fact that the Metaverse is an emerging area of research that is still in its infancy. That is to say, there is no established theoretical framework that gives a strong idea of what kind of themes to expect to find in the data, as with the deductive analytical technique. Accordingly, the intent is to develop a framework based on what can be found as themes that are not predetermined. Moreover, thematic analysis is more appropriate when analyzing and synthesizing a large body of literature—in the form of empirical studies, exploratory studies, conceptual frameworks, descriptive accounts, reviews, and so on. It can be used to produce complex conceptual cross–examinations of meanings in the qualitative data.

The main steps of this study’s methodology are as follows:

1. Review of literature of various disciplinarities that is related to data-driven smart urbanism. The aim is to deconstruct (“take apart”) a multidisciplinary text related to data-driven smart cities as a model of urbanism. The outcomes of this process are numerous themes, in this case “trends,” “processes,” “technologies,” “applications,” and “developments,” that are associated with this model of urbanism. It is important to be familiarized with all the aspects of the qualitative data collected. This step provides the foundation for the subsequent conceptual and critical analysis.
2. Recognizing patterns in seemingly random information (Boyatzis, 1998). The aim is to note major patterns and concepts within the results of the first step. The second step looks for similarities or patterns within the sample and then codes the results by concepts. Coding involves identifying passages of text that are linked by a common theme, allowing to index the text into categories and therefore establish a framework of thematic ideas about it. In this step, the preliminary codes identified are the features of data that appear meaningful and interesting, and the relevant data extracts are sorted according to the overarching themes. It is important to allude to the relationship between codes and themes.
3. Revising themes is about combining, separating, refining, or discarding initial themes. This relates to the inductive approach to thematic analysis. Data within the themes should cohere together meaningfully and be clear and identifiable as regards the distinction between these themes. A thematic map is generated from this step.
4. Identifying the digital and computing processes underlying the Metaverse as a virtual form of data-driven smart urbanism in terms of recognizing the specific and distinctive features of this model of urbanism.
5. Finding the theoretical relationships among the identified concepts and the Metaverse as a virtual form of data-driven smart urbanism—conceptualization.
6. Examining and discussing the risks and impacts of the identified digital and computing processes underlying the Metaverse as a virtual form of data-driven smart urbanism in the wake of the COVID-19 pandemic.
7. Transforming the analysis into an interpretable piece of writing by using vivid and compelling data extracts that relate to the overarching themes and literature. The outcome must go beyond a mere description of the preconceived themes and portray an analysis supported with evidence.

## Results

### The Escalating Trends and Processes Driving and Underlying the Metaverse as a Virtual Form of Data-Driven Smart Urbanism

The thematic analysis has identified five digital and computing processes repeated, which result from the digital transformation that both data-driven smart cities and the Metaverse immerse in through the processes of digitization and digitalization.

#### Digital Transformation: Digitization and Digitalization

We are moving into an era where digital instrumentation, digital hyper-connectivity, datafication, algorithmization, and platformization are routinely pervading the very fabric of urban ways of living thanks to the miniaturization of digital technologies as a set of machines, systems, and devices. Urban society is currently undergoing large-scale digital transformation in the light of both recent advances in science and technology and drastic shifts in governance. This extensive process of digitization and digitalization has been intensified, accelerated, and normalized by the COVID-19 pandemic. Digitization refers to the process of converting pieces of information, or encoding representations of urban actions, into a digital format that can be read, processed, transmitted, stored, re-used, and manipulated by computational systems for various use cases in the form of a series of zeroes and ones that describe a discrete set of points. Digitization is foundational in terms of making the connection between the physical world and computer software. It is an enabler for all the computational processes that generate value because of the need for manipulable and exploitable data. The process has exponentially increased the amount of data that could be further processed, analyzed, and harnessed. Digitalization is about the ways in which urban processes are organized through and around digital technologies. Generally, it entails facilitating and enhancing processes by leveraging digital technologies and digitized data with respect to productivity, efficiency, and effectiveness through taking a process from a human-driven series of events to software-driven series of events. In short, it is the use of digital technologies to advance processes and provide value-generating and maximizing opportunities. Changes associated with digitalization are applied to both data-driven smart cities as a social organization and the Metaverse as a commercial organization. They include distributed and flexible operational and functional processes and organizational arrangements, the automation and autonomy of administrative task systems, the adoption of solutionist and knowledge management systems, and communication and horizontal information platforms. In this context, digital transformation is urban transformation enabled by both digitization and digitalization, an integrated process which is necessary to pursue and spur innovation.

Data-driven smart cities represent an immersion in digital transformation, a process of digitization and digitalization that is enabled by the convergence of AI, the IoT, and Big Data and its far-reaching consequences— digital instrumentation, digital hyper-connectivity, datafication, algorithmization, and platformization (Bibri and Allam, 2022a; Calvo, 2020). These also pertain to the global architecture of computer mediation pertaining to the Metaverse as a virtual form of data-driven smart urbanism. Among the technological pillars of the Metaverse as a giant ecosystem application are user interactivity, XR, computer vision, AI/blockchain, robotics/IoT, edge cloud, wireless networks, and hardware infrastructure (Lee et al. 2021). Data-driven smart cities (e.g., Kaluarachchi, 2022; Sarker et al., 2020) are massively digitally instrumented and hyperconnected, intensively datafied, and increasingly algorithmized and platformized, and as such, they enable data-intensive, distributed computing across various urban domains based on innovative techniques, models, and decision support systems in the shape of large-scale data-driven AI systems. This is to enhance and optimize urban operations, functions, designs, strategies, and policies by means of generating “irreplaceable” values in the form of applied intelligence from monitoring, analyzing, and understanding citizens and places across different spatial scales and over different temporal scales. By the same token, at the heart of the Metaverse is a computational understanding of human users’ cognition, emotion, motivation, and behavior that reduces the experience of everyday life to logic and calculative rules and procedures (Bibri and Allam, 2022a). This implies that human users become more knowable and manageable and their behavior more predictable and controllable, thereby serving as passive data points feeding the AI and analytical systems that they have no interchange with or influence on. This relates to—as with smart urbanism—to quantitative universalism and reductionism, which refers to the socio-technical configurations that reduce urban phenomena into the purely quantitative (Bell, 2013; Haklay, 2013). Accordingly, the rich complexity of urban life is reduced to narrow quantitative and unitary languages, manifested in a plethora of platforms—with long-term implications for the wellness of citizens.

#### Platformization

Platform companies are becoming increasingly central to public and private life in urban society, transforming key urban sectors and domains of urban life. Many different types of platforms exist and vary across sectors, strategies, and practices. There has recently been a marked intensification of platformization in terms of a radical expansion and proliferation of platforms related to urban governance, urban economy, and urban services as key components of smart cities. As an ideological formation, platforms are associated with smart cities and sharing economy (Barta and Neff, 2016; Sadowski, 2020). The process of platformization deeply affects urban society—with socio-cultural, socio-political, and politico-economic consequences. The concept of “platformisation” has been derived from the notion of “platform.” Platforms combine digital technologies with organizational forms. Poell, Nieborg and van Dijck (Poell et al., 2019, p. 1) define platforms as “(re-)programmable digital infrastructures that facilitate and shape personalized interactions among end-users and complementors, organized through the systematic collection, algorithmic processing, monetization, and circulation of data.” Accordingly, they have been discussed in relation to the private, corporate, technology, and public sectors. Platformization refers to “the penetration of infrastructures, economic processes, and governmental frameworks of digital platforms in different economic sectors and spheres of life, as well as the reorganization of cultural practices and imaginations around these platforms” (Poell et al., 2019, p. 1). It entails the construction, operation, and exploitation of platforms and the alteration of existing organizational forms to align them with the logic of platforms (Casilli and Posada, 2019; Poell et al., 2019). In this network of agents, information, products, services, resources, and values are exchanged among companies, applications, users, and devices. Helmond’s (2015) defines platformization as the “penetration of platform extensions into the web, and the process in which third parties make their data platform-ready.” The computational infrastructures and informational resources involved in this process afford institutional relationships that are at the root of a platform’s evolution and growth as platforms provide a technological framework for other entities to use as a basis for further development (Helmond, 2015). Plantin et al. (2018) observe a simultaneous “platformisation of infrastructures” and “infrastructuralization of platforms”. The authors argue that digital technologies have made “possible lower cost, more dynamic, and more competitive alternatives to governmental or quasi-governmental monopoly infrastructures, in exchange for a transfer of wealth and responsibility to private enterprises” (Plantin et al., 2018, p. 306). Nieborg and Helmond (2019) analyse the case of Meta, where social media platforms are conceived as a “data infrastructure” that hosts a set of varied and constantly evolving “platform instances.” These instances are set to include many spheres of everyday life with the development of the Metaverse as a 3D network of numerous virtual worlds within the framework of visual cities thanks to the process of algorithmization and its key role in the dramatic shifts in the social organization resulting from social interactions and activities made possible by pairing digital data with connectivity to intensify datafication. There are many sets of platform instances pertaining to data-driven smart cities. One of them is associated with social infrastructure, which ties in well with the Metaverse in terms of its virtual services, as it typically involves assets that accommodate the social services provided by the public sector and related entities or through the financing of private provision of services. New digital technologies, interactive platforms, innovative solutions, and diverse forms of public-private cooperation have become of critical importance to overcome the social challenges and to bring about the needed transformations in a number of social domains. This is at the core of the assets of the social infrastructure of data-driven smart cities of the future, particularly in relation to citizen participation with respect to the following platform instances (Bibri and Krogstie, 2021):

- Crowdsourcing platforms to address important city issues related to different areas.
- Platform to enable citizens to influence their experience of the city by providing feedbacks and ratings.
- Platform where citizens can participate in the surveys organized by the city administration which can use the related data to adopt the resolutions in relation to the different domains of city life.
- Platform to engage more citizens in dialogue so as to gather input on their needs and demands, to evaluate their suggestions, and to identify and solve important issues.
- Platform to enable citizens to communicate as well as track the status and control the execution of their complaints related to city issues.
- Special portals to enable citizens to report the economic problems existing in the city in response to the adverse effects of pandemics and crises.
- Platforms to allow citizens to participate in urban technologies and policies, including:
  - Classrooms for learning about the uses and applications of and innovating in emerging digital technologies;
  - Entrepreneurial spaces for attracting startups and skilled innovators to create and promote new technologies;
  - Co-innovation centers for enabling close collaboration among different city stakeholders;
  - Participatory platforms for connecting city stakeholders to support decision-making processes; and Democracy platforms for enabling citizens to discuss government proposals as well as submit their own.

#### Algorithmization

Algorithmization has created the propensity for developing numerous platforms across various urban domains for a variety of practices and purposes. The ever-increasing trends towards the algorithmization of social interactions and human activities and the social organization resulting from these interactions and activities epitomize the core of the Metaverse vision. Algorithmization is the process of algorithmizing different urban activities and processes by converting their informal description into a set of well-defined instructions that can be used to perform a large-scale computation using mathematical and logical models for calculating specific functions, such as predicting a human user behavior, inferring a health or social status, augmenting a cognitive process, reading brain activities, and taking a decision on behalf of a human user. The AI and Big Data technologies, as a by-product of their normal operation, involve analyzing and interpreting massive amounts of data on citizens, places, and everyday objects to make decisions. The strong tendency to algorithmize the different areas of urban activity entails that AI algorithms take control of decision-making due to their perceived capacity of analysing constantly generated data, predicting the consequences of the decisions at play, and acting according to value maximisation criteria (Calvo, 2020, p. 1). Data-driven smart cities use numerous algorithmic tools and techniques to process the data collected from the monitoring of digital citizens and urban systems through extensive networks of data sources. This approach reduces the rich complexity of urban life and the unpredictability of urban systems to narrow quantitive and unitary languages. The reduced aspects embody cultural, ethical, social, and political values, nevertheless. Marked by functionalist visions, the fictional virtual cities depicted by the Metaverse tend to portray an algorithmic order, mirrored in the uniform functionalism of data-driven smart cities, where the fluidity, contingency, multidimensionality, complexity, and relationality of their systems, as well as the creativity, spontaneity, and emotionality of their citizens, are submitted to a techno-utopian fantasy of complete logical and calculative ordering.

#### Datafication

Agorithmizaton and platformization have been made possible by the marked intensification of the datafication of citizens and places in terms of the radical changes in the volume, granularity, heterogeneity, velocity, and veracity of the data being generated about every aspect of urban life thanks to digital hyper-connectivity and digital instrumentation. In other words, the instrumentation, datafication, and hyper-connectivity of the city have given rise to the process of algorithmizing and platformizing the different activities in the city. Datafication refers to the practice of taking a social activity, behavior, or process and turning it into meaningful data (Cukier and Mayer-Schöenberger, 2013), or to the act of transforming something into a quantified format (O’Neil and Schutt, 2013) so it can be structured, tabulated, and analysed (Cukier and Mayer-Schöenberger, 2013). As argued by (Cresswell, 2014) it is the datafication of the people and the geocoding of everything that are rendering data suddenly big. The processes of transforming social action into quantified data allows companies and government agencies to carry out monitoring and predictive analysis in real time of digital citizens via AI algorithms (van Dijck, 2014, 2016)—algorithmization. This implies that datafication is associated with data-driven AI analytics that permit more sophisticated mathematical and logical analyses to identify non-linear relationships among data for massive predictive analyses.

Smart cities are dependent upon their data to operate properly—and even to function at all with regard to almost all domains of urban life. In other words, smart city services and operational governance highly respond to a form of data-driven urbanism that reduces urban life to algorithmic rules and procedures (Kitchin, 2016) thanks to datafication. Smart cities are taking any possible quantifiable metric and squeezing value out of it for enhanced decision–making and deep insights pertaining to many domains of urban life. We generate enormous amounts of data on a daily basis, a binary trail of breadcrumbs that forms a map of urban life in terms of citizens’ experiences and urban dynamics, and the resulting disparate datasets can, if harnessed properly, open up a unique window of, and represent a goldmine, opportunity for making cities smarter and in tune with citizens’ actual needs and aspirations.

#### Digital Hyper-connectivity

Underlying the processes of algorthmization, platformization, and datafication is the digital hyper-connectivity of multiple systems, devices, things, and people. Related to the IoT, hyper-connectivity refers to the connectivity and interaction of everything that exist in digital environments, including systems, devices, objects, things, processes, activities, people, and data. With reference to smart cities, Calvo (2020, p. 141) describes digital hyper-connectivity as a three-pronged concept being:

- The trend towards the digital connectivity of everything
- The governance of the involved processes and the connected things enabled by the application of AI algorithms
- The AI algorithms fed through the data generation and analysis processes with “the objective, relevant information they need so they can make effective, efficient decisions capable of optimizing processes and making the behaviour of all the connected things in the system more predictable.”

The widespread diffusion of multiple wireless technologies, especially various 5G networks, will optimize and advance the sensing and collection of massive repositories of spatiotemporal data that represent society-wide proxies for human interactions and activities. The growing capabilities of 5G amounting to up to 10Gb (Lee et al., 2021) are providing new opportunities to the Metaverse as a giant ecosystem application that relies on the real-time transmission of colossal amounts of data. The increasing connectivity hinged on current 5G speeds and anticipated 6G speeds is expected to play a significant role in realizing the Metaverse vision (Allam et al. 2022b). Especially, it is expected that the Metaverse’s requirements will exceed 5G’s available bandwidth (Braud et al., 2020). The centrality of digitally enabled connectivity in understanding the consequences of the digitization, digitalization, and datafication is a product of two interrelated social constraints: (a) limited information processing abilities; and (b) visibility of data regardless of whether they are actively or willingly provided for decision making (Leonardi and Treem, 2020). This is at the core of the Metaverse as an organization whose reality is shaped and constrained by the finite limits of its ability to experience connectivity, regardless of the way it perceives the processes of data capture, storage, and representation as regards gaining detailed insights into human users due to the lack of producing representations of large, complex data. However, the global architecture of computer mediation underlying the Metaverse and its technical infrastructure connecting users have grown more robust. With this constant connectivity, the behaviors of people, organizations, and even technological devices and the real world are, by association, expected to be able to be visible (Flyverbom, 2019; Flyverbom et al., 2016).

**Fig. 1:**
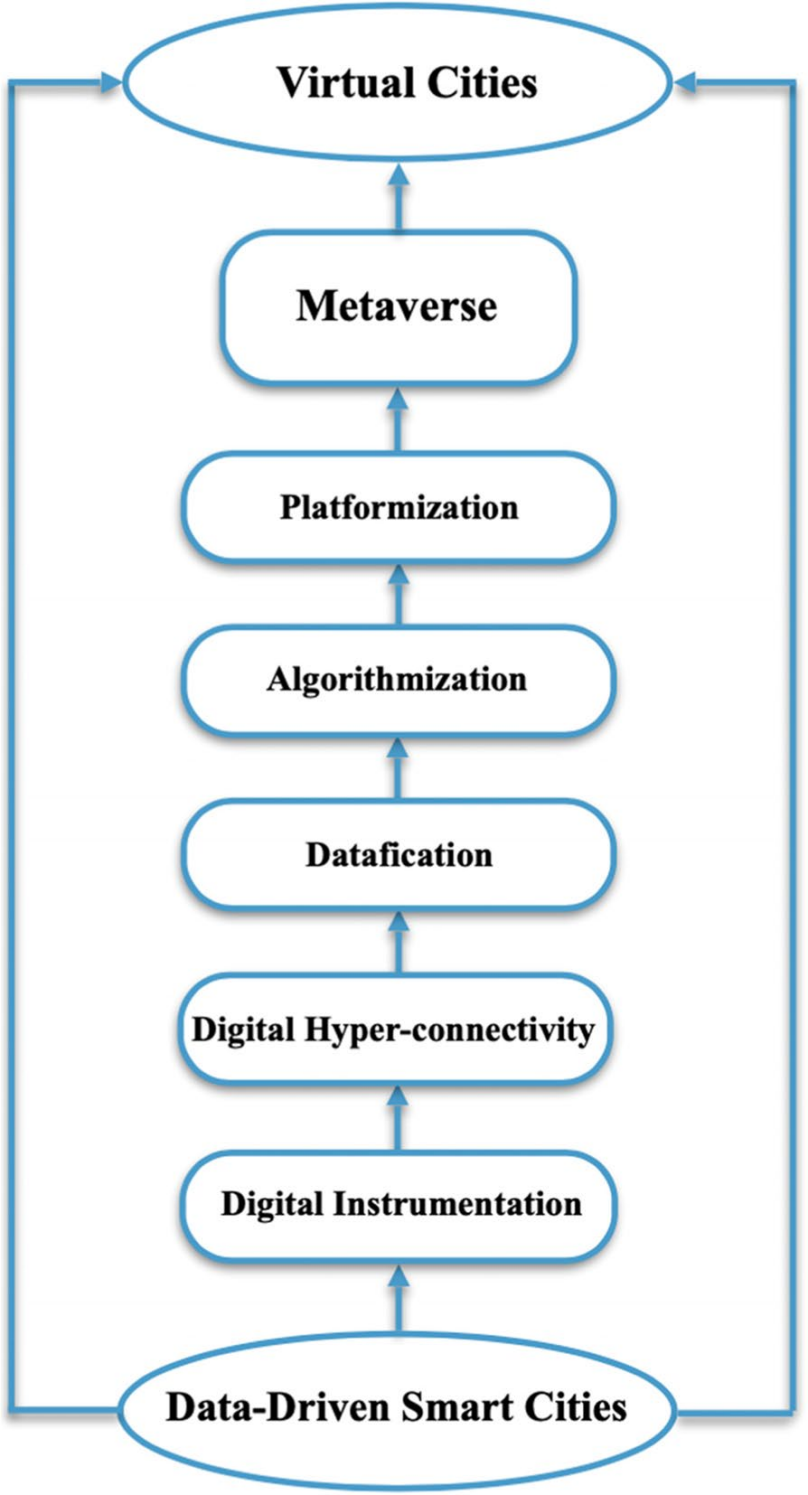
A conceptual framework for the digital and computing processes underlying the Metaverse as a virtual form of data-driven smart urbanism

#### Digital Instrumentation/Data Infrastructure

Digital instrumentation gives rise to hyper-connectivity and is aimed at producing big data via devices and data infrastructure, which in turn feed the collective tools, mechanisms, and instruments that transform the city into a data-driven enterprise. The latter is process of datafication is manifest in a variety of forms and can also be associated with the IoT and sensors as part of data infrastructure. This in turn relates to the information layer of data-driven smart cities, which involves the whole complex of data sources, including numerous types of sensors, cameras, transponders, meters, actuators, GPS, and transduction loops monitoring various phenomena, as well as a multitude of smartphone apps and sharing economy platforms generating a range of real-time location, movement, and activity data (Bibri and Krogstie, 2020b). These data are routinely generated about citizens and places by a range of private and public organizations. Smart cities are instrumented with digital devices and infrastructure that produce large amounts of data that enable real-time analysis of urban life and new modes of urban governance (Kitchin, 2014). Digital instrumentation is opening up dramatically different forms of the social organization (Batty et al., 2012) resulting from social interactions and activities, i.e., steering cities as well as controlling urban ways of living. It involves how data can be collected and analyzed, services can be organized and delivered, and operations can be streamlined. It is the domain of ICT companies themselves that are providing the detailed hardware and software of the operating system for emerging data-driven smart cities.

The data infrastructure and operating system for the city form what is called the horizontal information system for the city. The development of the data infrastructure has been captured through the notion of datafication: the ways in which digital platforms render into data, practices, and processes that elude quantification (Kitchin, 2014; Cukier and Mayer-Schöenberger, 2013; van Dijck, 2014; Mejias and Couldry, 2019). The operating system involves the tools used for storing, analyzing, and processing the data collected, as well as for interpreting these data, making forecasts on their basis, and identifying interconnection between different data ranges (Nikitin et al., 2016). The horizontal information system is one of the key components of the ICT infrastructure of data-driven smart cities with respect to performing the main functions of large-scale computation based on AI and big data analytics (Table 1) as well as linking together diverse smart technologies and solutions to coordinate city systems and domains.

**Table 1:** The key functions of the horizontal information system for data-driven smart cities

• Providing open platforms connecting the sensors installed and integrating the obtained sensed data • Aggregating and standardizing the flows of functional and territorial data from municipal sources, the systems of state control (mobility, energy, pollution level, etc.), business environment, and other state agencies (hospitals, cultural institutions, universities, schools, etc.), as well as from various surveillance (e.g., geosurveillance) technologies, for their subsequent integrated analysis and visualization in 3D format • Solving data disconnection problems through the open operating system that integrates and processes the information generated from urban sources • Reworking and repackaging the collected data for daily consumption by different stakeholders • Allowing the city authorities and third party users to gain access to the received data in a more structured and convenient manner for software development Integrating self-contained and unconnected solutions and the information systems used in the different functional departments of the city • Improving the efficiency and performance of applied technological solutions • Allowing the city authorities to take decisions on the optimization of urban activities on the short, medium, and long term basis.	

Source: Adapted from Bibri and Krogstie (2020a)

The quintessence of the idea of data-driven smart cities revolves around the necessity to coordinate and integrate technologies and their underlying processes that have clear synergies in their operation so that many new opportunities can be realized for strategic stakeholders through large-scale computation and platformization.

### A Conceptual Framework for the Digital and Computing Processes Underlying the Metaverse

The integrated framework illustrated in Fig. 1 is derived based on thematic analysis in terms of the identified core dimensions of the global architecture of computer mediation underlying the Metaverse as a virtual form of data-driven smart cities. It attempts to capture in a structured manner the underlying components of the digital and computing platform of the Metaverse. The basic idea revolves around the integration and combination of the same digital and computing processes enabling data-driven smart cities to build the Metaverse as a free-form design of virtually inhabitable cities. This is predicated on the assumption that speculative fiction plays an important role in shaping alternatives to the imaginaries of data-driven smart cities (Bina et al., 2020). The Metaverse seems to be edging closer to reality while paving the way for the emergence of virtual cities (Bibri, 2022), which, as found by Hemmati (2022), create more believable images than reality compared to real-world cities. This form of urban transformation has far-reaching implications for the way people will live in urban society—if the Metaverse is realized and deployed.

### The Risks and Impacts of the Metaverse as a Process of Platformization

Based on thematic analysis, the process of platformization—underpinned by digital instrumentation, digital hyper-connectivity, datafication, and algorithmization—is examined and discussed in terms of its risks to and impacts on urban society in the post-pandemic era.

#### Platformization: Institutional Dimensions and Social Implications

The practice and process of platformization has brought about a major digital transformation of the key sectors of urban society. This implies that institutional changes, cultural practices, digital technologies, and platforms are inextricably interrelated. There are many manifestations of this complex interplay, one of which is platform urbanism which has become central to the governance, economy, and experience of the city. Fields et al. (2020) provide insights into understanding the politics of platform urbanism. Caprotti et al. (2022) argue that platform urbanism as an evolution of the smart city is constituted by novel digitally enabled socio-technical assemblages that enable new forms of social, economic, and political intermediation and transaction. The increased datafication and algorithmization of social action facilitate new opportunities for organizing diverse forms of social organizations. Platforms constitute a key organizational strategy and operational logic of platform capitalism (Pasquale, 2016; Srnicek, 2017), digital capitalism (Faulkner-Gurstein and Wyatt, 2021, Wajcman 2015), surveillance capitalism (Zuboff 2019), and platform society (Van Dijck et al., 2018), all of which support dominant system of global capitalism. From a critical political economy perspective, platformization involves the process of intensifying the power and governance of global platform (Poell et al., 2019). Critical political economists have drawn attention to issues of surveillance and imperialism (Fuchs, 2017). Platforms are not politically neutral (Gillespie, 2010) and amplify the power of big tech companies that control them, creating new potentials for discipline and surveillance.

In light of the above, it is important to gain insights into how changes in the key institutional dimensions of platformization are intertwined in a complex interplay. These dimensions—data infrastructures, market relations, and governance frameworks—are simultaneously shaped by the (re-)organization of cultural practices around platforms as a result of platformization (Poell et al., 2019). Data infrastructures involve technologies and solutions that allow the collection and transfer of data for their further processing and analysis. Data handing as a resource for urban management and economies is a key feature of smart urbanism and platform urbanism. Both of these rely on pervasive and ubiquitous sensing and computing across digital urban spaces, as well as sophisticated analytics and advanced algorithms, thereby the centrality of data to the functioning of computing urban platforms. Data capture and usage are linked to “platform accumulation” in terms of deepening privatization, marketization, commodification, and consolidation forms pertaining to neoliberal capitalism (Meier and Manzerolle, 2018). In this respect, behavioral data collection is afforded by expanding platform infrastructures (Nieborg and Helmond, 2019) and their integration with a growing number of devices across many spheres of urban life. The myriad of the extensions pertaining to platformization allows platform operators, e.g., Meta, Google, Apple, and Microsoft, to transform virtually every instance of human social (inter-)action into data—datafication. This process is then algorithmized and haphazardly made available to a wide variety of external actors (Bucher, 2018; Langlois and Elmer, 2013). This connects well with the applications layer of data-driven smart cities that serves for the exchange of data among all the interested parties and the adoption of solutions based on the analysis of the collected data (Bibri and Krogstie, 2020b). This layer involves platforms with open data and tools of data visualization used for control over management system, automated systems of response to city-wide events, as well as a plethora of applications developed by city governments, state agencies, and other external developers.

Furthermore, market relations have significantly been shaped by the re-organization of cultural practices around platforms in terms of the means of society to communicate values and ways of living through social and behavioral interactions. This is due to the emergence of surveillance capitalism, which works by monitoring people's behaviors and movements online and in the physical world to capture their data for monetization, trading, and exploitation. Surveillance capitalism is one-sided claiming of the free raw material of private human experience for translation into behavioral data for profit and control (Zuboff, 2019). As a global platform, the Metaverse epitomizes the market-driven process of surveillance capitalism in terms of trading user personal information by translating it into behavioral data, relying on the mass surveillance of the Internet and thus scrutinizing online interactions, communications, and activities (Bibri and Allam, 2022b). These data are repackaged as prediction products with respect to what people will do now, soon, and in the future that are sold to behavioral futures markets—and offered to government elites. This repackaging is a multi-billion dollar industry consisting of a diverse ecosystem of different types of specialist companies as data brokers that are focused on specific markets (Kitchin, 2016). These companies offer services that are used to regulate, control, and govern end-users as well as the various systems and platforms with which they interact (Kitchin, 2014). Critical political economists argue that platform operators “are fully in charge of a platform’s techno-economic development” and therefore the power relations among platform operators, third parties, data brokers, and end-users are inherently asymmetrical (Poell et al., 2019). In particular, expanding the global market for new technological services and thus platforms often ignores their wider impacts on users and consumers. Therefore, consuming the Metaverse technologies must be approached carefully because big tech companies as centralized structures often have hidden, and are driven by, economic and political motives. Likewise, as argued by Viitanen and Kingston (2015), in smart city systems as a digital marketplace, citizen participation tends to be involuntary while the hegemony of big tech companies is inflated, resulting in a digital user experience with inherent biases and exclusionary issues.

In addition, platforms constitute increasingly complex multi-sided markets, and their arrangements in terms of aggregating transactions among a wide variety of end-users and third parties affect the distribution of economic power and wealth due to strong network effects (Poell et al., 2019). This pertains to platform intermediation in terms of how the relatively autonomous actors are convened and coordinated, and thus to platform capitalism as a process by which the intermediated network is seen a profit-making and investment channelling (Faulkner-Gurstein and Wyatt, 2021). As pointed out by Langley and Leyshon (2017), the processes of capitalization and the practices of intermediation are turned on by the generative force of the platform in digital economic circulation in a variety of ways. The Metaverse is attracting considerable investment, funding, and public attention and thereby giving rise to numerous R&D projects, programs, and consortia across a plethora of business and industry domains. It is pushing the global market towards unparalleled profitable paths. Meta and other platform providers, as well as major corporations, have begun investing billions of dollars to develop the Metaverse given the rising prospect that it will greatly impact urban society over the next decade. Bibri and Allam (2022b) discuss the financial gains and economic implications of the Metaverse in relation to immersive technologies. Johnson (2022) provides recent statistics and facts on the market capitalization of the Metaverse, Meta, and gaming worldwide. Lee et al. (2021) discuss in more detail the industry’s market structure of the Metaverse. In addition, as a digital twin of work in the physical world, the Metaverse platform will promote all kinds of brands. Given the rich diversity of technologies featured in the Metaverse and the broad variety of potential products and applications, it is believed that the economic prospects of the Metaverse will eventually justify current and future investments.

Platforms are becoming one of the key contemporary political–economic formations (Just, 2018; Vallas and Schor, 2020; Van Dijck et al., 2018) in terms of governance. Platforms steer both platform-based user interactions and economic transactions (Poell et al., 2019), which is associated with the governance dimension of platformization (Gillespie, 2018; Gorwa, 2019). This form of delegated governance represents a political approach to keeping platforms on task, where a larger framework of centralized power contains decentralized control and autonomy (Faulkner-Gurstein and Wyatt, 2021). Delegating control among actors is about exercising power over economic transactions by platforms, as apposed to hierarchies in terms of centralized power, markets as regards dispersed power, or networks as to parcelling power out to trusted collaborators (Vallas and Schor (2020). In this respect, structuring how end-users can interact with each other and other actors in the form of platform governance materializes through algorithmic sorting, thereby shaping what types of services become prominently visible and what remains largely out of sight (Bucher, 2018; Pasquale, 2015). Platforms govern through policies, which have to be agreed with when accessing platform’s services (van Dijck, 2013). On the basis of these terms and guidelines, platforms moderate what end-users can share and how they interact with each other (Gillespie, 2018), thereby conducting the actions and affairs of people with authority. This broadly relates to the exercise of “platform power” (Cohen, 2016). Within the Metaverse as a global platform, there are economic and political actors who exercise domination over others, which is associated with the politics of delegation and the politics of domination in terms of governance and government within platforms. However, there are often disputes and disagreements with local rules, regulatory frameworks, and social norms because platforms tend to use algorithms, interfaces, and policies as different governing instruments—without considering political and cultural traditions (Poell et al., 2019). Still, platformization is increasingly marked by strong state support and oversight (De Kloet et al., 2019) with respect to how this process is steered and managed by platform providers, such as Meta, Google, Apple, Microsoft, and Cisco, in collaboration with governments. This has become visible in the aftermath of the COVID-19 pandemic. As a way to help combat this pandemic, a number of companies are actively repurposing their platforms and data. Google and Apple are developing solutions to aid contact tracing via smartphones (Brandom and Robertson, 2020); Google is monitoring the effects of interventionist measures globally; and Meta, Apple, Google, and Microsoft are generating and storing real-time location and movement data while legitimating surveillance capitalism as well as invasively harvesting and exploiting personal (behavioral) data for profit-making (Kitchin, 2020).

#### The COVID-19 Crisis and the Ensuing Non-Spontaneous "Normality" of Social Order

The COVID-19 pandemic has forced new ways of living digitally in the urban world, drastically changing urban landscape in terms of the evolving urban patterns and the shifting nature of urban life. The abrupt digital transformation that has swept through the urban world in the aftermath of the COVID-19 pandemic, coupled with its disruptive impacts on people’s everyday life, seems to be in tandem with the envisioning process of the Metaverse in terms of its ultimate goal to datafy, algorithmize, and platformize urban ways of living towards virtual alternatives to the imaginaries of data-driven smart cities. Urban scholars have long explored fictional and imaginary representations of the city and urban life and their roles in shaping and framing urban change (Abbott, 2016; Bassett and Steinmueller, 2013; Dunn et al., 2014). However, the Metaverse was launched amid the COVID-19 pandemic, a crisis purported to be a rare opportunity that should be seized to reset and reimagine the urban world—though mainly in regard to its digital incarnation. Consequently, the “new normal” enforced by this crisis was nothing near to what—is termed in complex systems theory—“spontaneous order” or “self-organization.” The latter relates to the evolutionary resilience in the urban context (Davoudi et al., 2012), which denotes the ability of a complex system to not only bounce back from events causing a shock through robust behavior, but also to adapt and learn from the past behaviors to surpass the previous states by extending its capacity (Gunderson and Holling, 2002). As self–organizing social networks embedded in space and enabled by infrastructures, activities, and services (Bettencourt, 2014), cities are quintessential complex systems that exhibit unplanned order or self-organized behavior out of seemingly perceived chaos. Self–organization is created and controlled by no one. It results from human actions—not from human designs (Hayek, 1978) as the case of the “new normal” that is rather exhibited out of a chaos of another kind. Central to self–organization is that the actions of a group of individual constituents of a complex system are coordinated without centralized planning. This dynamical property of complex systems seems to be not characteristic of how urban society is bouncing back from the COVID-19 pandemic and adapting and learning from the past behaviors to surpass the previous pandemics. Historically, pandemics have been deeply impactful on the way cities have evolved, thereby forcing the agendas aligning with the prevalent narrative around the reset of urban society. In his work on the great reset and its impact on ways of living and working in cities as a result of the financial crisis of 2007-2008. Florida (2010) discusses how the past resets have shaped urban development, as well as what technological trends will emerge from the great reset. This work, which describes the future of cities, has been criticised for taking an overly elitist viewpoint by overstating the potential impact of the elite class and overlooking many socio-economic realities related to ways and choices of living.

The COVID-19 pandemic has served governments as a window of opportunity (Kingdon, 1984) to accelerate the development and adoption of big data technologies and thus digital transformation. Indeed, during this crisis, the world has braced for the “new normal,” where the use of advanced technologies have become mainstream and more embedded into almost every realm of urban society. As argued by Kitchin, 2020), the utility of the solutionist technologies deployed has been oversold, and this crisis served as an opportunity for governments to expand the roll-out and normalization of surveillance technologies, with no intention of rolling them back after the pandemic, and the “new normal” will include spatial sorting as to entering to public and private spaces. The systems deployed to combat the COVID-19 pandemic will become part of the “new normal” in monitoring and governing societies—and hence will not be turned off after the crisis (Sadowski, 2020; Stanley and Granick, 2020). In this respect, the state surveillance tends to “stick” when it is justified by pandemic or crisis events. The same technologies that have demanded fine-grained knowledge about movement, social networks, contact tracing, social distancing, and health status during the COVID-19 pandemic (Angwin 2020; Schwartz and Crocker, 2020; Stanley and Granick, 2020) will be utilized in the Metaverse as part of the global architecture of computer mediation upon which the implicit logic of surveillance capitalism depends.

The COVID-19 crisis seems to be laying the groundwork for shifting from data-driven smart urbanism to virtual platform urbanism. As concluded by Caprotti et al. (2022), there is a need to critically engage with platform urbanism in regard to its development in response to the COVID-19 pandemic, as well as how it may shape visions of the current and future reality in the city. As the imaginaries of smart cities have shown, the ways futures are imagined can frame and shape how urban societies and settlements evolve in their names (Bina et al., 2020). Fictional representations convey both “ future possibilities” and “warning signals” (Miles, 1990, 1993; Popper, 2009). With respect to the latter, the kind of digital transformation that is—accelerated by the COVID-19 pandemic—and reflected in the core vision of the Metaverse has been argued to be not for the better given the ethical, social, and political issues and risks it has raised. Since the onset of this crisis and its multifarious consequences have made it clear that its impact will not fade any time soon, and it will have a long-lasting impact on urban society and ways of living in it. Therefore, it has become of crucial importance to understand and find ways to address the risks and impacts of the rapid rollout of technologies across every sphere of urban society as regards technocracy, technocentricity, personal autonomy, freedom, privacy, cybersecurity, discrimination, and social exclusion, but to name a few (e.g., Allam, 2019, 2020, Aouragh et al. 2020; Calvo, 2020; Kitchin, 2020; Lee et al., 2020; McDonald, 2020; Stanley and Granick, 2020; Taeihagh, 2021; Taeihagh et al., 2021; Tan et al., 2021), These concerns are expected to exacerbate with the Metaverse (e.g., Bibri and Allam, 2022a, b; Gurov and Konkova, 2022; Falchuk et al., 2018; Lee et al., 2021; Rosenberg, 2022). This is predicated on the assumption that the magnitude of the data to be generated by the Metaverse will be far greater than that being collected from the Internet today due to the technical operational features of immersive technologies.

#### Data-Driven Corporate-led Technocratic Governance

The recent large-scale digital transformation of urban society has raised serious concerns and provoked disturbing questions about the core values of urban society being undermined or eroded. This situation has exacerbated the risks and other negative implications of smart urbanism and smart governance (Table 2). Emerging research within smart urbanism is increasingly investigating the associated empirical realities as they move from slick sales pitched by corporations to become new urban realities (Cowley et al., 2018; Cugurullo, 2017; Datta, 2015; Vanolo, 2016). In smart urbanism, citizens are managed and manipulated as a function of datasets in order to control urban governance and urban ways of living (Marvin et al., 2016). The data-driven smart urbanism model for sustainable development (Bibri, 2021c, 2021d) entrenches the idea that there are “no alternatives” to techno-managerialist governance of cities (Vanolo, 2014) by being promoted as optimizing and enhancing urban management through “standardized decision-making” (Joss 2016) that prioritizes efficiency over political action (Vanolo, 2014), which is seen as impediment (Bina et al., 2020). In data-driven governance, citizens play a “subaltern role” (Vanolo, 2016) and there is no real democratic participation (Hollands, 2015; Kitchin, 2014).

**Table 2:** The key issues and risks of smart urbanism and smart governance

**Smart Urbanism**	**Smart Governance**
(e.g., Bina et al., 2020; Cardullo & Kitchin, 2018; Kitchin, 2014, 2016; Marvin et al., 2016; Luque-Ayala & Marvin, 2015; Söderström & Paasche, 2014; Sadowski, 2016; Verrest & Pfeffer, 2019)	(e.g., Barns, 2018; Grossi et al., 2020; Grossi & Pianezzi, 2017; Grossi et al., 2020; Hollands, 2015; Kitchin, 2014; Sadowski & Pasquale, 2015; León & Rosen, 2020; McFarlane & Söderström, 2017; Pereira et al., 2018).
• Ignoring social, ethical political, cultural, economic, and historical contexts shaping urban life • Curtailing the opportunities for wider perspectives beyond technical systems and scientific processes • Lacking the acknowledgement that the urban is not confined to the administrative boundaries of the city • Overlooking local social-economic, cultural-political, and environmental contingencies in analyzing the development, implementation, and effects of urban policies • Marginalizing certain groups and creating multiple divides between those who have access to smart applications and those who do not • Reinforcing neoliberal economic growth, focusing on more affluent populations, and disempowering citizens • Breaking urban systems into pieces and reducing urban life to algorithmic processes to make the city knowable, manageable, and controllable • Pledging for sustainability as marketing strategy and overlooking sustainability concerns	• Concealing those urban issues, conflicts, and controversies that cannot be represented by digital models and embedded in data analytics techniques • Emphasizing the government as the prime initiator of innovative solutions and the private sector as their provider • Treating urban governance merely as a management problem that can be dealt with by making use of the power of big data analytics • Perceiving urban problems as being solvable primarily through the application of technologically derived knowledge • Neglecting the role of contextualization and place-based knowledge in shaping the process of governance • Focusing too much on the technical, engineering, and economic dimensions of urban governance while missing on the role of social processes in configuring its meaning in practice • Developing policies that are largely featured with the corporatization of urban governance • Resulting in highly unequal urban societies, characterized by unequal power relations, social exclusion, and unbalanced distributions of costs and benefits • Cementing surveillance practices and submitting spontaneity of choices to complete logical ordering.

Data-driven smart city systems “become a digital marketplace where citizen-consumers' participation is increasingly involuntary and…are defined through a digital consumer experience that has inherent biases and leaves parts of the city and its population unaccounted for. This renders the city less resilient in the face of future social…risks” (Viitanen & Kingston, 2015). Paradoxically, ubiquitous citizen sensing and computing across digital urban spaces are often represented as a way of enabling progressive citizen empowerment as part of e-government or smart governance. Focusing on the relationships between ICT-enabled citizen-government collaboration and social sustainability and how contextual circumstances influence these related elements, Tomor et al. (2019) found that empirical evidence for the alleged benefits in this regard is sparse, and the emerging picture is ambiguous as it reports both positive and negative effects regarding the achievements of smart governance. One of the conclusions drawn by the authors is that smart governance, in the sense of ICT-enabled government-citizen collaboration, is still rare. Despite the increasing variety of collaboration-based digital instruments, a one-way information supply in citizen–government interactions tends to dominate. Although governments promote online citizen engagement and civic empowerment, they do not encourage deliberation or any broad-based public–civil interactions in practice. Urban affairs are framed in socio-political configurations of technocratic regimes and constituted in social constructions of big data systems as an apolitical or neutral matter, respectively, an illusion of political neutrality and objective view of smart technologies (Bibri, 2022; Söderström et al., 2014).

The Metaverse as a techno-urban utopia is built on the monitoring of citizens and places through extensive networks of data collection, processed and analyzed via AI algorithms and mathematical models. Mathematics presents an answer to a set of pre-defined variables, which is why it appears “rational,” the algorithmic rules are made up to get a certain outcome. Algorithmic governance involves unevenness and inequity which reproduce data justice issues (Dencik et al., 2016; Taylor, 2017) across different demographics (Benjamin, 2019; Noble, 2018) with potentially harmful consequences. The quantified, digitally stored, manipulatable, and shared information on users and consumers is seen as a key source of exploitable, investable value (Faulkner-Gurstein & Wyatt, 2021). Regardless, technocratic governance replaces democratic policy-making and politics and data-driven AI systems replace wider urban knowledge and expertise (Chandler, 2015; Söderström et al., 2014). Urban life is far more than digital imprisonment. Democracy is subordinated to the governmental and corporate elites who control smart technologies and govern “by code” (Söderström et al., 2014, p. 315). Outsourcing democratic resilience increase the power of the powerful elites, raising further concerns over accountability, representation, and transparency (Bibri & Allam, 2022b). Regardless, the Metaverse will be a digital marketplace where the supremacy and dominance of big tech companies will be further inflated, and its numerous virtual worlds will be defined through the experience of human users that will reinforce control and deepen inequality and social exclusion. This renders the Metaverse way less equitable, inclusive, and safe than data-driven smart cities in the face of future vulnerabilities and risks. Overemphasising advanced computing and immersive technologies in the context of data-driven smart cities is more likely to undermine social and ethical values (Allam 2020, Allam and Dhunny 2019, Allam et al. 2022). As argued by Bina et al. (2020, p. 8) “fictional representations powerfully explore the dystopian consequences of the dream of dominium over nature and the resultant production of extreme, oppressive, and unstable environments, animating and extending a range of warning that social scientific critique often touches upon”.

#### Governmentality

The consequences of massively deploying surveillance technologies during the COVID-19 pandemic have been argued to have significant downstream effects that are to be suffered by citizens. This event has already affected urban ways of living and the way citizens self-govern themselves drastically. Kitchin (2020) questions the technical and practical efficacy of surveillance technologies and examines their implications for governmentality. This concept denotes how people govern themselves (Foucault, 1991) or exercise government “beyond the state” (Rose & Miller, 1992). As a term combining government and rationality, govenmentality represents the tactics of government that allow it to define and redefine what competencies it entails, or the calculated means that allow it to shape, guide, or affect the conduct of people. Accordingly, the state designs systems for defining populations, including management and administration mechanisms and ways of classifying individuals or groups based on certain norms, which make them known and visible by means of their identification, categorization, and control (Foucault, 1977). Kitchin (2020) outlines an agenda for documenting how surveillance technologies unfold in practice and impact on governmentality. The promotion and use of invasive technologies in the age of surveillance capitalism trump concerns over civil liveries. Routinizing new forms of social and spatial sorting as a result of the new type of management enabled by surveillance technologies has “the potential to permanently shift the nature of governmentality and to also act as a pathway towards authoritarian forms of governance where technology is used to actively impose the will of the state onto citizens” (Kitchin, 2020).

Numerous investigations have demonstrated that states have a poor record when it comes to practicing dataveillance (Lyon, 2015) and geosurveillance, which lend a legitimacy to authoritarianism concerns. Dataveillance entails the systematic surveillance of people's activities and behaviors on the Internet. Monitoring and investigating the digital data pertaining to personal details and online and virtual interactions, actions, and communications will be the primary purpose of the creation and use of data in the Metaverse. Geosurveillance is the tracking and tracing of location and movement of people, vehicles, goods, objects, products, and services and the monitoring of interactions and relationships across space and time. With the event of the COVID-19 crisis, technologies beyond smartphone infrastructure, such as the IoT, AI systems, Big Data ecosystems, Edge Computing, XR, Blockchain are being subject to control creep, i.e., their original purpose is being extended to perform mass surveillance in order to normalize and cement the new biopolitical architecture of urban society. Central to the biopolitics (Foucault, 1977) of the COVID-19 pandemic is to control bodies and their movement and to trace their contact. Being thoroughly spatial in regard to its articulation, it regulates public and private spaces and spatial access and behavior, as well as generates particular forms of spatiality (Kitchin, 2020). With reference to the practice of governmental surveillance, Crampton (2003) argues that surveillance and security operate by establishing norms that assess risks and threats, which entails deploying geosurveillance in response to dangerousness and subjecting people to management as at-risk resources. This practice relates to what Foucault (1977) calls a “governmental society,” which operates at the level of populations and their distribution across territory. The technocratic, algorithmic, automated nature of technologies can shift the governmental logic from surveillance and discipline to capture and control (Deleuze 1992). Given the long-lasting impact of the COVID-19 pandemic, urban ways of living will be intimately and permanently interwoven with data-driven governmentality. Therefore, it is important to critically engage with how this form of platformization may create alternatives to the imaginary of data-driven smart cities based on the fictional representations of future urban worlds imagined by the Metaverse.

#### Privacy, Security, and Trust

The concern about privacy is part of a larger concern about control, about people having control over their own lives. This contradicts the logic of surveillance capitalism, which underpins platform society—where platforms have penetrated the core of urban society, affecting civic and public practices and democratic and ethical values. The responsibility of “anchoring public values and the common good in a platform society,” including privacy, security, and safety, as well as fairness, control, and accountability (Van Dijck et al., 2018) is increasingly being outsourced to the global technology sector. Platformized surveillance is at the heart of data-driven smart cities and thus the Metaverse. With respect to the former, Calvo (2020) addresses the moral implications of the hyper-connectivity, datafication, and algorithmization of urban society within the ethical realm of smart cities. Kitchin (2016) examines the ethics of smart cities, focusing on privacy, datafication, dataveillance, and geosurveillance. The author argues that smart city initiatives need to be re-cast in ways that adopt ethical principles designed to realize the benefits of smart cities while reducing pernicious effects. Drawing on this study, Bibri and Allam (2022a) examine the forms, practices, and ethics of the Metaverse as a virtual form of data-driven smart cities, paying particular attention to: privacy, dataveillance, and geosurveillance, among others. The authors highlight the ethical implications the Metaverse will have on the experience of everyday life in post-pandemic urban society. They argue that the Metaverse will do more harm than good to human users due to the massive misuse of the hyper-connectivity, datafication, algorithmization, and platformization underlying the global architecture of computer mediation upon which surveillance capitalism depends. However, privacy threats are worrying most of the users and consumers of the Metaverse, as the privacy–enhancing mechanisms proposed thus far remain inadequate to solve this ethical conundrum. In reality, technology can only safeguard privacy, and even this potential is associated with inherent limitations and embedded flaws. Thus, privacy is a real challenge and quandary facing the Metaverse (e.g., Acquisti et al., 2011, Acquisti et al., 2014; Dick, 2020; Falchuk et al., 2018; Lee et al., 2021; Leenes, 2007), especially in relation to face recognition and edge computing.

Not only the issue of privacy but also the issues of security, trust, and accountability have long been, and continue to be, a subject of much debate and an area of intensive research (e.g., Alqubaisi et al., 2020; Mollah et al., 2017; Boddington., 2021; Cuzzocrea, 2014; Lee et al., 2021; Liu et al., 2015; Haber, 2020; Ouda et al., 2010; Ryan 2011). Based on recent statistics published by Johnson (2022), among the concerns posed by the Metaverse are, in addition to privacy, hacking, trust, data abuse, and identity protection. Lee et al. (2021) provide a detailed discussion on security, trust, and accountability, as well as privacy, in the context of the Metaverse. While much of ongoing debate revolve around acceptable practices in regard to accessing and disclosing personal and sensitive information about people, the era of Artificial Intelligence of Things (AIoT) marks the end of privacy. What is risky to the users of the Metaverse is the idea that this platform will be steered and controlled by big data companies—considering the aggressive tactics and engagement strategies that are currently being used in social media platforms for malicious purposes. The risks to the users of the Metaverse can not be mitigated or solved by changing the business models of platform providers or by establishing strong industry norms among them (Rosenberg, 2022). The idea of platforms being seen as black boxes emanates from the proprietary nature of AI algorithms, corporate ownership, and control and thus the concerns about scrutiny, accountability and transparency, and addressing them reflects a limited and limiting horizon and potential of socio-political solutions. It follows that the Metaverse will most likely employ new deceptive methods based on opaque and largely invisible algorithms to impede the ability of people to grasp their ethical and societal implications, as well as to keep them unaware at best and ignorant at worst of the kind of arrangements that are intricately interwoven with governmental apparatuses and their techniques (Bibri & Allam, 2022b). Technologies that are designed to deliver specific services are enrolled into policing and security apparatuses (Kitchin, 2020). With reference to social media platforms, Fuchs (2017) found that the surveillance capitalism fuses with the surveillance state. This issue is further complicated by hidden collaborative arrangements with state security apparatuses (Zuboff, 2019). It follows that the regulatory frameworks that control dataveillance and geosurveillance as main reasons for privacy harms are most likely not to be enacted by or enforced on big tech companies due to their vested interests with other large corporations and government agencies.

#### Data Governance

Data governance is a complex and slippery concept, especially when it comes to its implementation as a set of decisions, and in different settings. It relates to the political dimension of Internet governance and international relations, that is, the governing of cross-border data flows based on a whole system of policies, practices, and institutions managing various types of data. Policies are a set of laws, rules, regulations, norms, and actions adopted by governments and mediated by civic and public institutions. This entails the formation and utilization of networks for linking data between civic institutions across urban society. Data governance refers to the institutional systems that manage the processes of storing, processing, analyzing, using, sharing, transacting, and trading data by or in the name of the government (Bonina & Eaton, 2020). In data governance, efficiencies tend to be prioritized over regulatory requirements and user and consumer services. In the wake of the COVID-19 pandemic, many countries have enacted policies to govern data processes to serve efficiency and effectiveness at the expense of privacy, equity, and safety due to the accelerated rollout of digital technologies to combat it. Li et al. (2022) explore the extent to which the COVID-19 pandemic has led to policy change in data governance and the implications of such change for the post-COVID-19 era. The resulting extensive use and accelerated development of digital technologies associated with the collection and utilization of personal and sensitive data have raised and intensify concerns regarding data governance, data privacy, and data security as a whole (Parker et al., 2020). This is due to the fact that big data companies determine the current research in the field of data governance, and this has implications for developing and implementing regulatory frameworks for data governance across many domains of urban society. The development and use of data tools for containing and controlling the COVID-19 pandemic have proven to have a long-lasting detrimental impact on urban society and data governance, enacting new and changing existing policies to achieve the “new goals” of data-driven smart cities. To put it differently, this crisis has exacerbated the issues of the increasing involvement of big data companies in data policy and data privacy through the accelerated adoption of big data technologies (Li et al., 2022). Therefore, there is a need to critically investigate new power geometries of corporate, legal, and regulatory alignments with respect to platform urbanism (Caprotti et al., 2022), virtual platform urbanism (Bibri, 2022), and platform society (Van Dijck et al., 2018) with respect to data governance and data privacy.

Data privacy measures and mechanisms have been a subject of much debate since the early 1990s, as well as of a great deal of activity in legislatures. This has resulted in “data protection oversight agencies and a modest level of jurisprudence” in many countries, while provisions that enable dataveillance and geosurveillance are voluminous (Clarke and Greenleaf 2017). Oversight decisions are largely influenced by the lobbying of big tech companies while insisting their evolving technology is too complex and fast-moving to be regulated. Regardless, personal data cannot be defined based on privacy regulations alone, as these tend to lag behind technological innovations due to their rapid pace, thereby the need to develop a principled framework that keeps up with them as regards what personal data mean. In this respect, Rosenberg (2022) propose some of the regulatory solutions to mitigate the risks of the Metaverse, including restricting monitoring, emotional analysis, virtual product placements, and simulated personas. The author argues that government and industry actors must consider aggressive regulations promptly, predicated on the assumption that it would become difficult to unwind them if the problems are embedded in the business models and digital infrastructure of the Metaverse. However, it is unfeasible to enact these regulatory solutions— considering the current reality of social media platforms where AI-based algorithms are designed to serve devious purposes, although there are possibilities to implement the privacy-by-design approach and its principles.

In addition, many governments have repealed privacy protection to enable the widespread use of personal data as a means to tackle the COVID-19 pandemic, and this will become part of the “new normal” and hence will not be revoked again after this crisis. This is just like all the systems deployed to combat the COVID-19 pandemic (Sadowski, 2020). Thus, the COVID-19 pandemic will have profound impacts on the future of data governance, the new measures will have lasting effects on data privacy, and personal and sensitive data will be shared across the different platforms of urban society. The core values of society are globally the very stakes in the struggle over its platformization, including disputes and disagreements over regulation between platform providers and city councils and power clashes between global markets and (supra-)national governments (Van Dijck et al., 2018).

## Discussion and Conclusion

This study analysed the emerging trends enabling and driving data-driven smart cities based on a thematic analysis approach in order to derive a conceptual framework for the digital and computing processes underlying the Metaverse as a virtual form of data-driven smart urbanism. These processes are: digital instrumentation, digital hyper-connectivity, datafication, algorithmization, and platformization. They are inextricably interrelated in that they shape and build on one another at the technical and operational levels towards enabling the functioning of the Metaverse and the future urban world it imagines. The proposed framework represents a conceptual structure intended to serve as a guide for building a model of virtual urbanism that can expand the structure into something useful on the basis of further in-depth qualitative analyses, empirical investigations, and practical implementations.

Further, this study examined and discussed the risks and impacts of the digital and computing processes underlying the Metaverse as a virtual form of data-driven smart urbanism, paying particular attention to: platformization; the COVID-19 crisis and the ensuing non-spontaneous "normality" of social order; data-driven corporate-led technocratic governance; governmentality; privacy, security, and trust; and data governance. This study argues that the digital and computing processes—as intricately interwoven with the entirety of urban ways of living—arouse contention and controversy due to their negative effects on civic and public practices and participatory and democratic processes. Due to the inherent ethical and societal implications of science and technology, more explicit democratic processes are needed for enhancing civic participation in the shaping of the Metaverse as a form of scientific and technological development. The ultimate goal is to structure such development in ways that are collectively the most democratically beneficial for urban society. The concerns over long-term data privacy and data governance as a result of the wide deployment of big data technologies may remain unabated, but it is necessary to devise concrete institutional measures and practices pertaining to platformization in order to address and overcome these concerns. Otherwise, they may lead to the deterioration of the quality of the governance of urban society as a whole in the post-pandemic COVID-19 era, which would hinder future government efforts to gain citizen trust and encourage citizen cooperation. In other words, citizen distrust in government could be exacerbated if the development and use of big data technologies are not carefully implemented to respond to citizen needs. The Metaverse raises critical concerns about the governance of urban society due to the logic of surveillance capitalism and what constitutes the global architecture of computer mediation it depends on with regard to the underlying mechanisms that are designed to increase the power of the powerful (corporate and government elites) and undermine the public values of urban society. As summarized by Zuboff (2019), surveillance capitalism is best described "as a coup from above, not an overthrow of the state but rather an overthrow of the people's sovereignty and a prominent force in the perilous drift towards democratic de-consolidation that now threatens Western liberal democracies” (Gray, 2019). Surveillance capitalism leads to democratic backsliding, privacy loss, and freedom erosion. Large corporations have often been at the forefront of debates over such practices (Rikap & Lundvall, 2020), often criticised for not re-assessing the process and practice of platformization. Therefore, governments in democracies must employ new approaches when regulating long-lasting big data technologies and their escalating rate and scale of use based on deep analysis to avoid unexpected and potentially disastrous or lethal consequences in the long run.

By looking closely at the institutional dimensions of platformisation, it becomes clear how this multifaceted process and practice brings about a large-scale digital transformation of the spheres of urban society, why it raises serious concerns over the underlying mechanisms, and what challenges it presents for strategic actors. It is crucial to gain insights into how changes in the dimensions of platformization may shape one another—but rather in a mutual process. In this regard, future endeavors need to focus on finding ways to regulate the Metaverse as a global process and practice of platformization democratically, ethically, and effectively through relevant social structures and institutions while understanding the key underlying mechanisms at work. One of the key challenges to address in this regard is to integrate platforms in urban society without undermining cultural features, such as norms, beliefs, and values, and without increasing disparities in the distribution of benefits and costs and of wealth and power. This is a worthy scholarly endeavor in itself, so is the extent to which a deeper understanding of the mechanisms at play will bring concrete changes to the functioning of the Metaverse. In addition, a holistic philosophical and analytical framework needs to be developed and applied to enhance the understanding of how political and institutional changes are entangled with shifting socio-cultural practices as a result of the emerging socially, politically, and economically oriented platforms and vice versa. The framework of Science, Technology, and Society (STS) can bring new insights into the ever-evolving dynamics and increasing complexity of platformization. At the core of this framework is a systemic exploration of the ways in which different forms of science and technology emerge and evolve and become institutionalized and socially anchored—interwoven with policy and politics and thus globally disseminated, as well as of the risks and impacts of science and technology (Bibri, 2022). This framework is essentially applied to investigate science and technology in its wider social context (e.g., Biagioli, 1999; Hess, 1997; Jasanoff et al., 1995; Sismondo, 2004). Indeed, a systemic inquiry into the relationships between the institutional and social dimensions of platformization as a form of scientific and technological development is of crucial importance because it will bring into view the tensions between the Metaverse and institutional practices and governance frameworks.

## Data Availability

Not applicable
